# Transactions Ohio State Dental Society

**Published:** 1877-05

**Authors:** 


### TRANSACTIONS.

The transactions of the Ohio State Dental Society, has just been issued. This is a beautifully gotten up book of 102 pages, containing the proceedings, papers and discussion of that body at its last meeting; they are interesting and valuable. It contains in addition the revised Constitution and By-laws, and a list of the membership of the Society, corrected for this issue.

In addition to these will be found, a list of the dentists of the state of Ohio, as complete as it could be made by sending into every county for a report of the dentists therein. In nearly all of these, the list is correct: there are a few instances in which omissions may exist. In a very few instances the reports did not contain the name of an immediate competitor of the reporter; such little streaks of human nature will sometimes come to the surface. The list is sufficiently accurate for all practicable purposes. The volume can be obtained by application to Ransom & Randolph, Toledo, Ohio, or to the editor of the Register. They are free—without money and without price.

